# Supplementary figures and images for: Integrative Analysis of Hepatopancreas Transcriptome and Proteome in Female Eriocheir sinensis under Thermal Stress
Source: Int J Mol Sci. 2024 Jun 30;25(13):7249. doi: 10.3390/ijms25137249 (PMC11242792; doi:10.3390/ijms25137249)

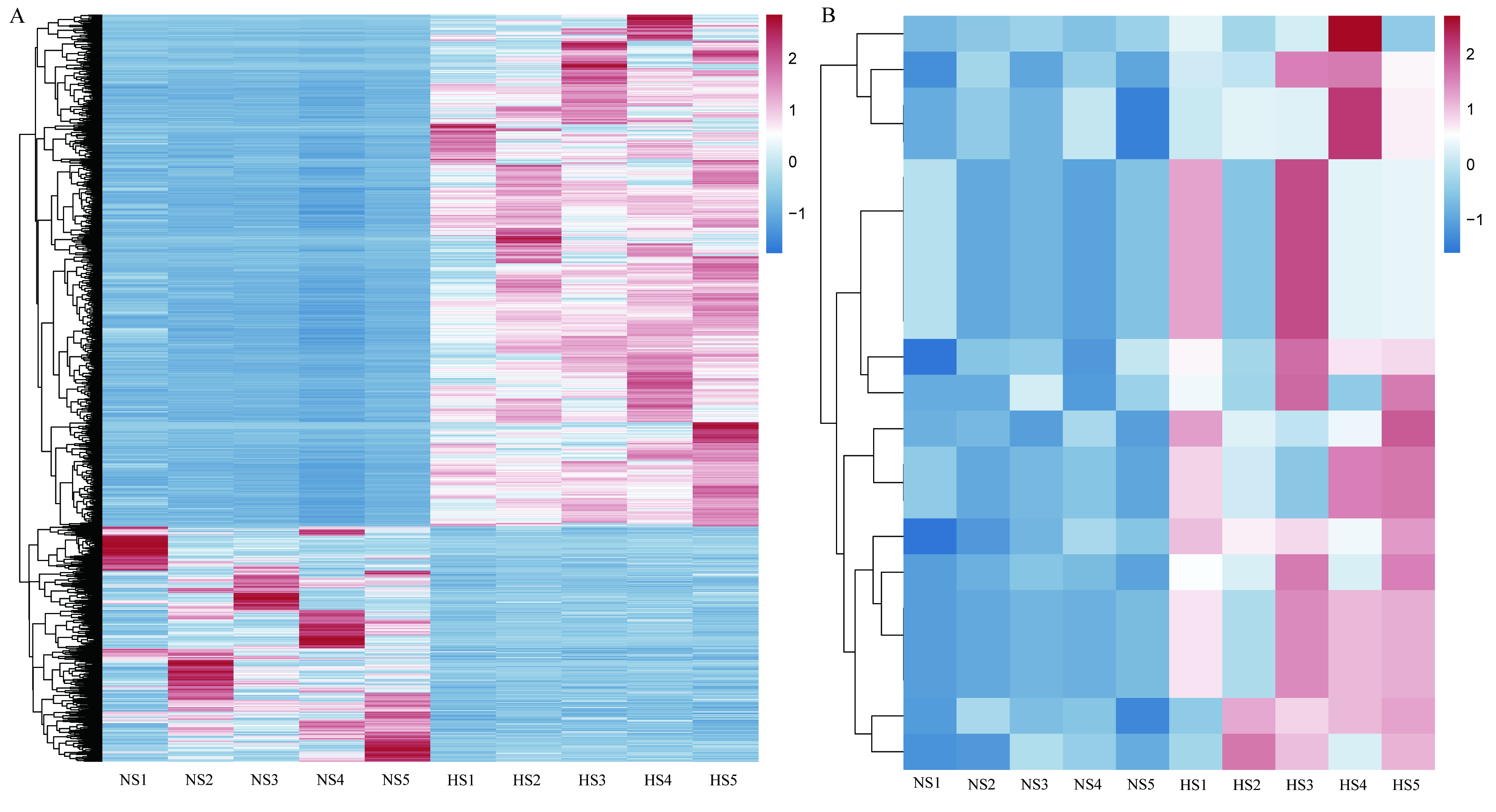

Supplement: Supplementary file 1 [file ijms-25-07249-s001.zip › ijms-2989780-supplementary (2).tif]
